# Rare-Earth-Doped Barium Molybdate Up-Conversion Phosphor with Potential Application in Optical Temperature Sensing

**DOI:** 10.3390/ma15227917

**Published:** 2022-11-09

**Authors:** Jung-Hyun Wi, Sang-Geon Park, Young-Seok Shim, Kwangjae Lee, Jae-Yong Jung

**Affiliations:** 1 Department of Smart Manufacturing Engineering, Changwon National University, Changwon 51140, Korea; 2 Department of Mechatronics Convergence Engineering, Changwon National University, Changwon 51140, Korea; 3 School of Energy, Materials and Chemical Engineering, Korea University of Technology and Education, Cheonan 31253, Korea; 4 Department of Information Security Engineering, SangMyung University, Hongjimum 2-gil, Seoul 03016, Korea; 5 Research and Business Development Foundation, Engineering Building, Silla University, Busan 45985, Korea

**Keywords:** up-conversion, phosphors, BaMoO_4_, sensing, co-precipitation

## Abstract

A BaMoO_4_:[Er^3+^]/[Yb^3+^] up-conversion (UC) phosphor was synthesized by co-precipitation and calcination of the precursor at 800 °C. The main peak (112) for the synthesized phosphor was strongly detected in the XRD pattern and had a tetragonal structure. The doping of rare-earth ions affected the crystal lattice by shifting the main peak, decreasing the lattice constant, and shifting the position of the Raman signal. The synthesized upconverted phosphor exhibited strong green signals at 530 and 553 nm and weak red signals at 657 nm when excited at 980 nm. The green light emission intensity of the UC phosphor increased as the pump power of the laser increased due to the two-photon effect. The synthesized upconverted phosphor was prepared as a pellet and flexible composite. Thermal quenching led to a decrease in luminescence intensity as the temperature increased, which means that the phosphor can be applied to optical temperature sensing.

## 1. Introduction

Oxidizing compounds, including silicate, borate, aluminate, and molybdate, exhibit strong absorption in the ultraviolet region and are thus known as phosphor materials doped with rare-earth ions. Among them, molybdate is considered a good host crystal for light-emitting materials because it has a scheelite-type crystal structure and exhibits excellent chemical and thermal stability [1,2,3,4]. It has been used in a wide range of fields, such as phosphor materials doped with rare-earth ions using barium molybdate (BaMoO_4_) as a host and in LEDs, lasers, optical fibers, and catalysts [5,6]. BaMoO_4_ phosphors show strong absorbance in the ultraviolet region because they are doped with rare-earth ions such as terbium (Tb^3+^), europium (Eu^3+^), dysprosium (Dy^3+^), and samarium (Sm^3+^), and many studies have reported green and red phosphors in the visible region [7,8,9,10]. Jung et al. reported the fabrication of a flexible composite that can be applied to LEDs by observing the change in the luminescence characteristics based on the doping concentration of Tb^3+^ rare-earth ions using BaMoO_4_ as a host [11]. Lee et al. synthesized a white luminescent BaMoO_4_ phosphor by co-doping Dy^3+^ and Sm^3+^ rare-earth ions at room temperature via co-precipitation and reported the change in luminescence characteristics based on the concentration of Sm^3+^ ions [12]. In contrast to down-conversion phosphors that absorb strong light energy such as UV light and emit light in the visible region, up-conversion (UC) phosphors that absorb weak light energy in the infrared region and emit light in the visible region have also attracted attention [13,14]. They can be used in various fields such as bio-imaging, security, and energy harvesting in addition to display and lighting [15,16]. Rai et al. synthesized a BaMoO_4_:Pr^3+^ UC phosphor doped with praseodymium (Pr^3+^) rare-earth ions using a solid-state reaction method. The synthesized phosphor was excited with a diode laser at 808 nm to observe the emission spectrum in the 450–725 nm region [17]. Chung et al. synthesized BaMoO_4_ UC phosphors co-doped with erbium (Er^3+^) and ytterbium (Yb^3+^) rare-earth ions using the citrate gel method. The synthesized phosphor showed strong green emission at 530 and 550 nm and weak red emission at 660 nm when excited with a 980 nm laser. In addition, changes in the UC luminescence characteristics based on the excitation luminescence intensity were observed [18]. In this study, a UC phosphor was synthesized by preparing a BaMoO_4_ precursor doped with rare-earth ions ([Er^3+^]/[Yb^3+^]) using a co-precipitation method and heat treatment. In the present study, the structure and luminescence characteristics of the synthesized phosphor were investigated. The luminescence characteristics of the synthesized phosphor varied with the excitation energy power and temperature, suggesting that it could be used as a temperature sensor.

## 2. Materials and Methods

### 2.1. Synthesis of BaMoO_4_:[Er^3+^]/[Yb^3+^] UC Phosphor

**Figure 1:**
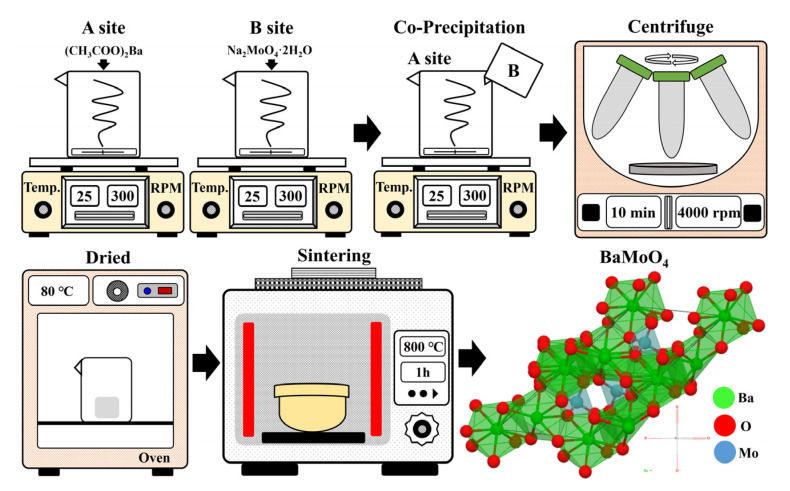
Experimental procedure for preparing the BaMoO_4_:[Er^3+^]/[Yb^3+^] up-conversion phosphors.

Barium acetate ((CH_3_CO_2_)_2_Ba, Sigma-Aldrich, St. Louis, MO, USA), sodium molybdate dihydrate (Na_2_MoO_4_·2H_2_O, Sigma-Aldrich, St. Louis, MO, USA), ytterbium (III) nitrate pentahydrate (Yb(NO_3_)_3_·5H_2_O, Yb^3+^, St. Louis, MO, USA), and erbium(III) nitrate pentahydrate (Er(NO_3_)_3_·5H_2_O, Er^3+^, St. Louis, MO, USA) were used as starting materials. Two solutions were prepared in separate beakers. Solution ‘A’ was prepared by dissolving (CH_3_CO_2_)_2_Ba (10 mmol) in 50 mL of distilled water. Solution ‘B’ was prepared by dissolving Na_2_MoO_4_·2H_2_O (10 mmol) in 50 mL of distilled water (Figure 1). Once the solutions were completely dissolved, So-lution ‘B’ was slowly poured into the beaker containing Solution ‘A’. The mixture was stirred for approximately 10 min. Subsequently, the powder was recovered using a centrifuge (4000 rpm, 5 min), rinsed with distilled water two times to remove residual sodium, and then placed in a drying oven at 80 °C for 24 h. The UC phosphor was synthe-sized with BaMoO_4_ as a host. The precursor was prepared by simultaneously adding Yb(NO_3_)_3_·5H_2_O and Er(NO_3_)_3_·5H_2_O to Solution “A”. The prepared precursor was sintered at 800 °C for 1 h to produce BaMoO4:[Er^3+^]/[ Yb^3+^] phosphor. The concentration of rare-earth [RE] ions of Yb^3+^ was kept constant at 0.5 mmol, while the concentration of Er^3+^ added was varied ([Er^3+^]/[Yb^3+^] ~ 0.1, 0.2, 0.3, 0.4, 0.6, 0.8, 1) [19,20,21,22]. The number of moles of the reagent used and the amount of doped rare earth added are detailed in Table 1.

### 2.2. Characterization

TThe crystal structure of the synthesized phosphor powder was determined using an X-ray diffraction apparatus (XRD, X’Pert PRO MPD, 40 kV, 30mA) with CuKα radiation (wavelength: 1.5406 Å) at a scan rate of 4°/min and a diffraction angle of 10 to 70°. Field-emission scanning electron microscopy (FESEM) was used to characterize the size, microscopic surface, and shape of the crystal grains (FESEM, Brono, CZ, MIRA I LMH, TESCAN). A semicon-ductor pulse laser (TCLDM9, Thorlabs, Jessup, MD, USA) with an emission output of 100 mW at an excitation wavelength of 980 nm and a spectrometer (HR4000, Ocean Optics, Ostfildern, Germany) connected with a photomultiplier was used to measure the fluores-cence spectrum by UC and the emission spectrum. Raman spectroscopy (JP/NRS-3300, 532 nm, 100 mW solid-state primary laser) was mainly employed to understand the fluorescence mechanism of UC. The energy absorption and energy transfer processes in the excited state were analyzed by varying the intensity of the pulsed laser and meas-uring the changes in the fluorescence intensity using Raman spectroscopy.

### 2.3. Fabricated Pellet and Flexible Composite

The synthesized phosphor powder and an aqueous solution of 1 mL of polyvinyl alcohol (PVA, 10 wt.%, Sigma-Aldrich, St. Louis, MO, USA) were kneaded with a pestle and bowl. Pellets were prepared by placing them in a 1-inch mold and applying a pressure of 200 MPa. A flexible composite was prepared by mixing 0.1 g of the synthesized phosphor powder and 1 g of polydimethylsiloxane, pouring it into a square mold, and subsequently curing it in an oven at 80 °C for 1 h.

## 3. Results and Discussion

### 3.1. Structure and Surface Morphology of BaMoO_4_ UC Phosphor

**Figure 2:**
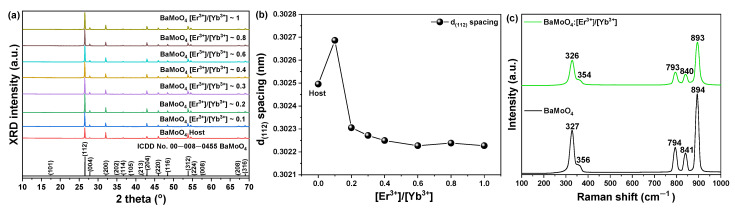
(**a**) XRD patterns of synthesized BaMoO_4_ and BaMoO_4_:[Er^3+^]/[Yb^3+^] powders, and (**b**) change in d_(112)_ spacing and (**c**) Raman spectra of BaMoO_4_ (black line) and BaMoO_4_:[Er^3+^]/[Yb^3+^] (green line) under 532 nm laser.

XRD analysis was performed to investigate the change in the crystal structure of the BaMoO_4_ UC phosphor, which was synthesized by calcining the precursor prepared by the co-precipitation method at 800 °C, based on the rare-earth doping. The synthesized UC phosphor had a tetragonal (a = 5.580 Å, b = 5.580 Å, c = 12.821 Å, JCPDS Card No. 00-008-0455) structure irrespective of the rare-earth doping, and a (112) signal was detected corresponding to the main peak in the XRD pattern (Figure 2a). Except for the main peaks, (004), (200), (114), (204), (220), (116), (312), and (224) peaks were weakly detected. When the amount of BaMoO_4_ UC phosphor synthesized by doping with rare-earth elements was BaMoO_4_:[Er^3+^]/Yb^3+^] ~ 0.1 (0.3027 nm), the lattice constant of the main peak (112), calculated using Bragg’s equation [23], increased compared to that of the undoped host (0.3025 nm), as shown in Figure 2b. Because of the doping of the rare-earth element with a relatively large ionic radius, the primary peak shifted and the lattice constant (Ba^2+^ = 1.35 Å, Yb^3+^ = 0.868 Å, Er^3+^ = 0.89 Å) changed. Lovisa et al. synthesized a ZnMoO_4_ phosphor co-doped with Tb^3+^ and Pr^3+^ using a sonochemical method. In ZnMoO_4_, the primary peak (120) shifted, and the lattice constant changed because of the doped rare-earth element. In this study, it was reported that the change was caused by rare-earth elements with relatively large ionic radii [24].

Raman spectra were obtained by excitation of the sample with a 532 nm laser to observe the molecular frequency change caused by the rare-earth ions located in the doped BaMoO_4_ crystal lattice. The synthesized BaMoO_4_ had frequencies of 328, 362, 793, 840, and 893 cm^−1^. Figure S1 shows a slight shift in the position of frequencies in BaMoO_4_:[Er^3+^]/[Yb^3+^] samples doped with rare-earth ions. According to research studies, the vibrations of molecules are affected by the energy transferred from the outside by doping them with rare-earth ions [25].

**Figure 3:**
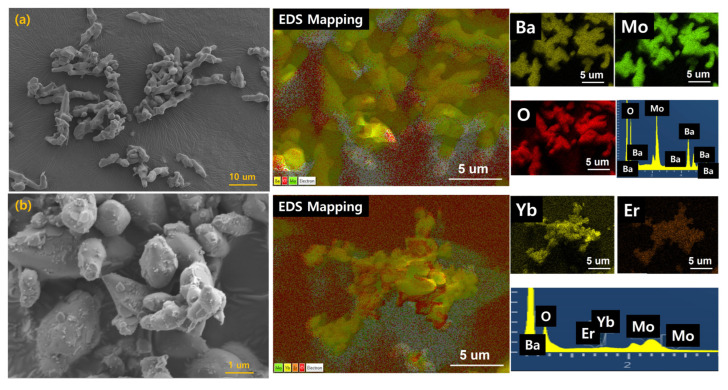
FE-SEM image and EDS Mapping analysis of (**a**) BaMoO_4_ and (**b**) BaMoO_4_:[Er^3+^]/[Yb^3+^] powder.

The synthesized BaMoO_4_ had a size of about 11 μm in the longitudinal direction and about 3.4 μm in the transverse direction and a sharp shape. Rare-earth-doped BaMoO_4_:[Er^3+^]/[Yb^3+^] had an elliptical particle shape with a size of about 3 μm in the longitudinal direction and about 1.3 μm in the transverse direction, as shown in Figure 3. In the EDS analysis, the components of Ba, Mo, and O and the rare-earth Yb and Er were detected through mapping, and the doped components could be confirmed (Figure S2).

### 3.2. Luminescence Properties of BaMoO_4_ UC Phosphor

**Figure 4:**
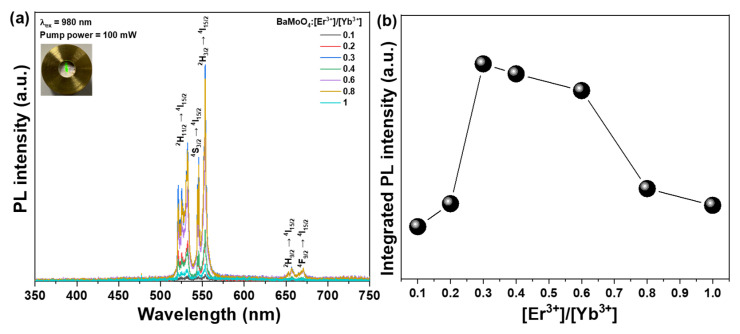
(**a**) PL spectra, and (**b**) change in PL intensity based on the Er^3+^ concentration under 980 nm at 100 mW in the BaMoO_4_:[Er^3+^]/[Yb^3+^] up-conversion phosphors.

Figure 4a shows the photoluminescence (PL) spectrum and intensity changes according to the amount of Er^3+^ ions added to the BaMoO_4_ UC phosphor co-doped with Er^3+^ and Yb^3+^ rare-earth ions. In the UC phosphor powder excited with a semiconductor laser at 980 nm, strong green peaks at 530 and 553 nm and slightly weak red peaks at 657 nm were observed. The synthesized UC phosphor absorbed light energy at a wavelength of 980 nm emitted from the semiconductor laser by Yb^3+^ ions and showed UC properties by the excitation of Er^3+^ ions through energy transfer by another photon and absorption of the excited state [26]. The green emission (^2^H_11/2_ → ^4^I_15/2_, ^4^S_3/2_ → ^4^I_15/2_, and ^2^H_3/2_ → ^4^I_15/2_) was observed by the UC PL spectrum generated by the excitation pumping light due to the transition of Er^3+^ ions [27].

**Figure 5:**
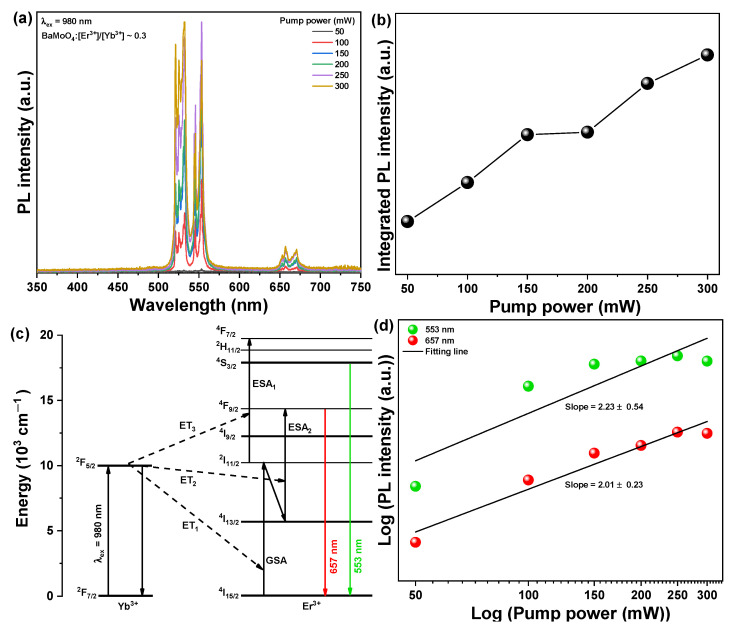
(**a**) PL spectra; (**b**) Change in integrated PL intensity; (**c**) Schematic energy transfer process; and (**d**) Linear fitting at 553 and 657 nm intensity according to pump power under 980 nm of BaMoO_4_:[Er^3+^]/[Yb^3+^] ~0.3 up-conversion phosphors.

The intensity of emission increased as the doping concentration of Er^3+^ ions increased, with the strongest emission peak observed at [Er^3+^]/[Yb^3+^] ~ 0. However, when the doping concentration was further increased, the concentration of Er^3+^ ions became too high, resulting in decreased luminescence intensity owing to the cross-relaxation process between Er^3+^ ions and the concentration quenching phenomenon [28]. Furthermore, energy loss occurred when energy was transferred back to the Yb^3+^ ions distributed around the Er^3+^ ions, indicating that the light emission intensity decreases as a result of the upward conversion process (Figure 4b). The UC emission characteristic change was observed for the [Er^3+^]/[Yb^3+^] ~ 0.3 sample based on the intensity of the excitation pumping light source. The sample obtained the strongest emission intensity by changing the Er^3+^ concentration and fixing the intensity of the excitation pumping light source at 100 mW, as shown in Figure 5. The intensity of the UC PL increased when the intensity of the excitation light source at 980 nm was changed from 50 to 300 mW, as shown in Figure 5a,b. The UC process causes an energy transfer (ET) process in which the light energy absorbed by Yb^3+^ ions is transferred to Er^3+^ ions in addition to an excited state absorption (ESA) process by an additional energy transfer of the excited Er^3+^ ions (Figure 5c). The excitation process from the ^4^I_11/2_ level to the ^4^F_7/2_ level for green emissions is related to the following three processes [29]:

ESA: ^4^I_11/2_ + photon (980 nm) → ^4^F_7/2_ (1)

ET: ^2^F_5/2_(Yb^3+^) + ^4^I_11/2_ (Er^3+^) → ^2^F_7/2_(Yb^3+^) + ^4^F_7/2_(Er^3+^) (2)

CR (cross-relaxation): ^4^I_11/2_(Er^3+^) + ^4^I_11/2_(Er^3+^) → ^4^F_7/2_(Er^3+^) + ^4^I_15/2_(Er^3+^) (3)

The ESA process occurs for a single ion, whereas the ET process occurs when two ions are involved. The BaMoO_4_ UC phosphor doped with Er^3+^ and Yb^3+^ ions absorbed photons of excitation wavelength at 980 nm, and subsequently, Yb^3+^ ions at the ^2^F_7/2_ level were excited to the ^2^F_5/2_ level. The excited Yb^3+^ ions further excited the Er^3+^ ions to the 4I_11/2_ level, which then returned to the ground state to the adjacent Er^3+^ ions through the ET process (^2^F_5/2_ (Yb^3+^) + ^4^I_15/2_ (Er^3+^) → ^2^F_7/2_ (Yb^3+^) + ^4^I_11/2_ (Er^3+)^). When pumping with excitation light of wavelength 980 nm, the first step involves excitation of Er^3+^ ions to ^4^I_11/2_ level through ET_1_ and GSA processes. The lifetime of the ^4^I_11/2_ level was long, and the electrons were occupied at the ^4^F_7/2_ level of the Er^3+^ ion by the ET_3_ and ESA_1_ processes (^2^F_5/2_ (Yb^3+^) + ^4^I_11/2_ (Er^3+^) → ^2^F_7/2_ (Yb^3+^) + ^4^F_7/2_ (Er^3+^)) as a result of the excitation of the Yb^3+^ ion through the absorption of another photon. Another mechanism by which electrons could be occupied at the ^4^F_7/2_ level involves the cross-relaxation process between the adjacent Er^3+^ ions, in which one of the two Er^3+^ ions at the ^4^I_11/2_ level interacts with the other to gain energy and move to the ^4^F_7/2_ level. The other loses energy and transitions to the ^4^I_15/2_ level in the ground state. Using this mechanism, green light was emitted at 530 nm (^2^H_11/2_ → ^4^I_15/2_) and at 553 nm (^4^S_3/2_ → ^4^I_15/2_). In red fluorescence via UC, electrons undergo the ET_2_ and ESA_2_ processes and are occupied at the ^4^I_11/2_ level by a non-radiative transition from the ^4^I_13/2_ level. Red light at 657 nm wavelength (^4^F_9/2_ → ^4^I_15/2_) was emitted in the process of ESA_2_ after the ^4^F_9/2_ level was occupied by electrons [30,31]. The intensity of fluorescence (*I*) emitted by the UC is proportional to the intensity of fluorescence of the pump excitation light (*P*), which can be expressed by the following Equation [32]:

(4) $$I_{vis}\;\propto \;P^{n}$$

where *I_vis_* is the up-conversion emission intensity, *P* is the intensity of the excitation light (mW), and *n* is the number of absorbed photons required to be excited at the emission level. Figure 5c shows the fitting of the fluorescence intensity of the PL spectrum obtained by changing the intensity of the excitation pumping light of the BaMoO_4_:[Er^3+^]/[Yb^3+^] ~ 0.3 UC phosphor. The slope of the green fluorescence peak at 553 nm changed according to the pump excitation light, which was estimated to be approximately 2.23. The green fluorescence emission was due to a two-photon process that involved two excitation photons. The slope of the red fluorescence peak at 657 nm was estimated to be approximately 2.01. This phenomenon occurs when an electron in the ground state absorbs the first photon, is excited to the ^4^I_11/2_ level, occupies the ^4^I_13/2_ level by nonradiative transition, and occupies the ^4^F (^4^F_9/2_ → ^4^I_15/2_) level through the ESA_2_ process, which absorbs the second photon as the ET_2_ process. This is a two-photon process [33]. 

**Figure 6:**
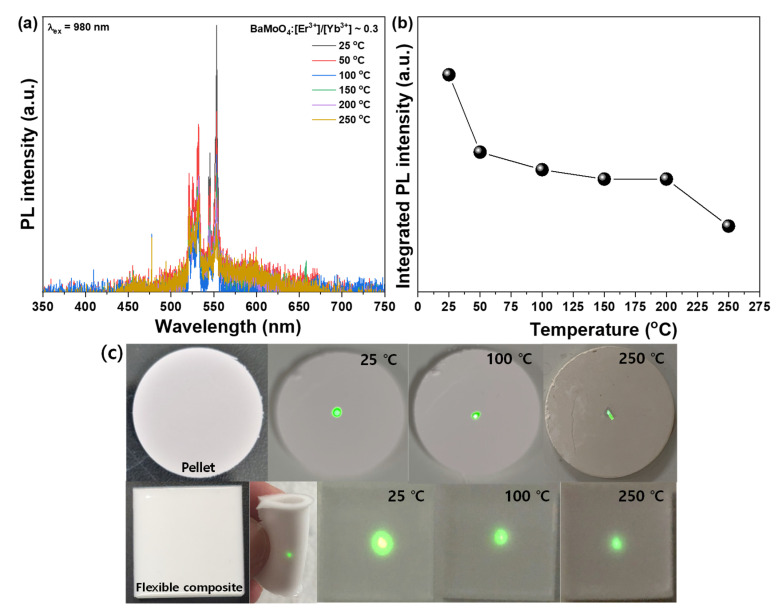
(**a**) PL spectra of changing temperature; (**b**) Integrated PL intensity; and (**c**) Photograph of pellet and flexible composite under 980 nm at 100 mW of BaMoO_4_:[Er^3+^]/[Yb^3+^] ~0.3 up-conversion phosphors.

The PL spectrum of the characteristic change in UC fluorescence with temperature is shown in Figure 6a,b. The intensity of UC PL decreased when the temperature was raised from room temperature to 250 °C. In the process where Yb^3+^ ions absorb the light energy of the excitation-pumping light source and transfer it to Er^3+^ ions, the host BaMoO_4_ does not transmit energy well due to the lattice vibration caused by heat. This thermal quenching expands the crystal lattice of the host and reduces the luminescence properties of the UC phosphor. Liao et al. reported that, as the temperature increased, the change in UC luminescence characteristics was positive, while the thermal expansion of the host was negative [34]. This phenomenon was attributed to the radiative trapping of Yb^3+^ because the lattice shrinkage reduces the distance of Yb^3+^/Er^3+^ at high temperatures and promotes the radiative trapping of Yb^3+^. In phosphorescent materials co-doped with Yb^3+^/Er^3+^, Yb^3+^ both acts as a radiation trap to store energy as well as a sensitizer to transfer energy to Er^3+^. These radiation traps may have promoted the release of Er^3+^ ions. In addition, the Yb^3+/^Er^3+^ distance decreases as the temperature increases. Generally, the ET process between the sensitizer (Yb^3+^) and activator (Er^3+^) is caused by dipole interactions. Since ET efficiency is proportional to r^-6^ (where r is the donor-acceptor distance), then we can infer that increasing the temperature can substantially improve the ET efficiency [34]. The BaMoO_4_:[Er^3+^]/[Yb^3+^] UC phosphor synthesized in this study exhibited a decrease in UC emission characteristics owing to the positive thermal expansion of the host by heat supplied from the outside. Sylwia et al. synthesized a UC phosphor doped with Er^3+^ ions using SrF_2_ as a host. As in this study, it was shown that the UC luminescence properties decreased as the temperature increased. The temperature-dependent green luminescence quenching related to intensified nonradiative relaxation processes at higher temperatures reported. The decrease in UC luminescence properties due to temperature increase is related to thermal expansion of the host as well as temperature-dependent lattice vibration-induced phonons that quenches the UC emission [35]. As shown in The UC phosphor powder was molded into pellets and heated directly as shown in Figure 6c. The sample in contact with the excitation light source at 980 nm showed green light emission. The decrease in the size of the emitted dot as the temperature increased at room temperature was visible to the naked eye. The present study suggests that the synthesized BaMoO_4_ UC phosphor can be used as a temperature sensor owing to its characteristics.

## 4. Conclusions

BaMoO_4_:[Er^3+^]/[Yb^3+^] up-conversion phosphors applicable to optical temperature sensing were synthesized by co-precipitation and calcination at 800 °C. The synthesized phosphor powder exhibited a tetragonal structure in the XRD analysis, and the main peak (112) phase was clearly observed. In addition, the position of the peak shifted as detected by rare-earth doping with a relatively large ion radius, and the lattice constant decreased. It was further confirmed that the Raman signal caused a slight change caused by rare-earth doping and influenced the crystal lattice. When excited by a 980 nm laser, the synthesized BaMoO_4_:[Er^3+^]/[Yb^3+^] phosphor emitted a strong green light, and the intensity of the light emission changed as the laser pump power increased. In addition, the luminescence intensity increases as the temperature decreases. This phenomenon was attributed to the two-photon process of the excited-state absorption process and the energy-transfer process. The synthesized phosphor was prepared as a pellet and flexible composite. It was observed that the luminescence intensity of the pellet and composite varied with temperature. It is therefore suggested that the synthesized phosphor can be used as temperature sensors.

## Figures and Tables

**Table 1:** Reagents and moles used in the synthesis.

BaMoO_4_ Up-Conversion Phosphor Synthesis				
Reagents	(CH_3_COO)_2_Ba	Na_2_MoO_4_·2H_2_O	Yb(NO_3_)_3_·5H_2_O	Er(NO_3_)_3_·5H_2_O
Molecular Weight (g/mol)	255.42	241.95	449.13	443.35
Used mole (mmol)	10	10	0.5	0.05~0.5
[Er^3+^]/[Yb^3+^] Ratio				
Reagents	(CH_3_COO)_2_Ba	Na_2_MoO_4_·2H_2_O	Yb(NO_3_)_3_·5H_2_O	Er(NO_3_)_3_·5H_2_O
Used mole (mmol)	10	10	0.5	0.05
	10	10	0.5	0.1
	10	10	0.5	0.15
	10	10	0.5	0.2
	10	10	0.5	0.3
	10	10	0.5	0.4
	10	10	0.5	0.5

## Data Availability

The data presented in this study are available on request from the corresponding author.

## Supplementary Materials

The following supporting information can be downloaded at: https://www.mdpi.com/article/10.3390/ma15227917/s1, Figure S1: Raman spectra of (a) BaMoO_4_ and (b) BaMoO_4_:[Er^3+^]/[Yb^3+^] under 532 nm laser; Figure S2: FE-SEM EDS mapping analysis of (a) BaMoO4 and (b) BaMoO4:[Er3+]/[Yb3+] under 532 nm laser.
