# Supplementary material for: Rare-Earth-Doped Barium Molybdate Up-Conversion Phosphor with Potential Application in Optical Temperature Sensing
Source: Materials (Basel). 2022 Nov 9;15(22):7917. doi: 10.3390/ma15227917 (PMC9697691; doi:10.3390/ma15227917)
Supplement: Supplementary file 1 [file materials-15-07917-s001.zip › materials-1999700-supplementary.pdf]

materials

MDPI

Article

Rare-Earth-Doped Barium Molybdate Up-Conversion Phosphor with Potential Application in Optical Temperature Sensing

Jung-Hyun Wi [1], Sang-Geon Park [2,*], Young-Seok Shim [3], Kwangjae Lee [4] and Jae-Yong Jung [5,*]

1 Department of Smart Manufacturing Engineering, Changwon National University, Changwon 51140, Korea
2 Department of Mechatronics Convergence Engineering, Changwon National University, Changwon 51140, Korea
3 School of Energy, Materials and Chemical Engineering, Korea University of Technology and Education, Cheonan 31253, Korea
4 Department of Information Security Engineering, SangMyung University, Hongjimum 2-gil, Seoul 03016, Korea
5 Research and Business Development Foundation, Engineering Building, Silla University, Busan 45985, Korea
* Correspondence: sgpark@changwon.ac.kr (S.-G.P.); eayoung21@naver.com (J.-Y.J.); Tel.: +82-55-213-3845 (S.-G.P.); +82-51-999-6441 (J.-Y.J.)

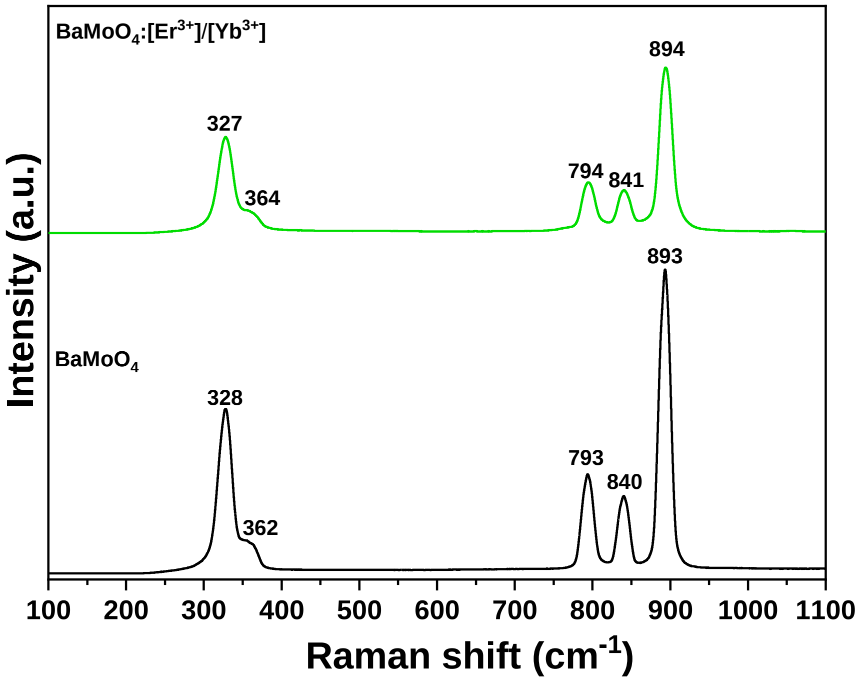

Figure S1. Raman spectra of (a) $BaMoO_4$ and (b) $BaMoO_4$:[Er^{3+}]/[Yb^{3+}] under 532 nm laser.

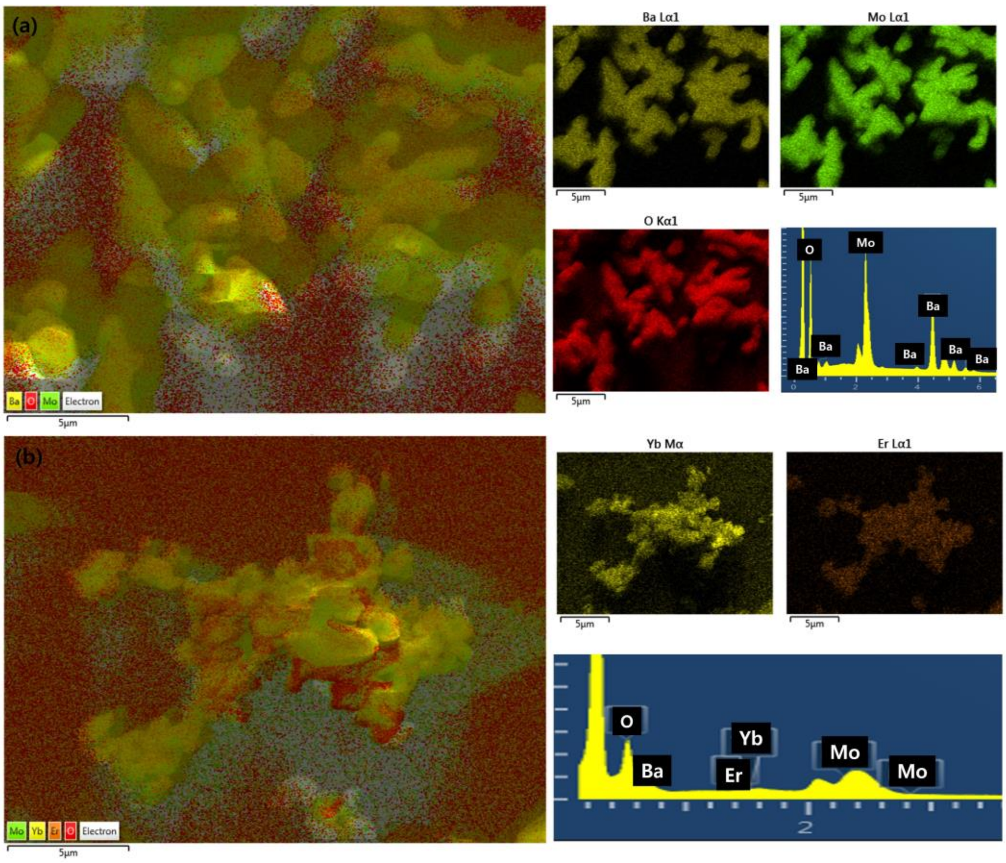

Figure S2. FE-SEM EDS mapping analysis of (**a**) $BaMoO_4$ and (**b**) $BaMoO_4$:[Er^{3+}]/[Yb^{3+}] under 532 nm laser.
